# The Cyclooxygenase 2 Inhibitor Etoricoxib as Adjunctive Therapy in Tuberculosis Impairs Macrophage Control of Mycobacterial Growth

**DOI:** 10.1093/infdis/jiad390

**Published:** 2023-09-18

**Authors:** Kristin G Nore, Claire Louet, Marit Bugge, Alexandre Gidon, Marthe Jøntvedt Jørgensen, Synne Jenum, Anne Ma Dyrhol-Riise, Kristian Tonby, Trude Helen Flo

**Affiliations:** Institute of Clinical Medicine, Faculty of Medicine, University of Oslo, Oslo, Norway; Department of Infectious Diseases, Oslo University Hospital, Oslo, Norway; Centre of Molecular Inflammation Research, Department of Clinical and Molecular Medicine, Norwegian University of Science and Technology, Trondheim, Norway; Centre of Molecular Inflammation Research, Department of Clinical and Molecular Medicine, Norwegian University of Science and Technology, Trondheim, Norway; Centre of Molecular Inflammation Research, Department of Clinical and Molecular Medicine, Norwegian University of Science and Technology, Trondheim, Norway; Institute of Clinical Medicine, Faculty of Medicine, University of Oslo, Oslo, Norway; Department of Infectious Diseases, Oslo University Hospital, Oslo, Norway; Institute of Clinical Medicine, Faculty of Medicine, University of Oslo, Oslo, Norway; Department of Infectious Diseases, Oslo University Hospital, Oslo, Norway; Institute of Clinical Medicine, Faculty of Medicine, University of Oslo, Oslo, Norway; Department of Infectious Diseases, Oslo University Hospital, Oslo, Norway; Centre of Molecular Inflammation Research, Department of Clinical and Molecular Medicine, Norwegian University of Science and Technology, Trondheim, Norway; Department of Infection, St Olav's Hospital, Trondheim, Norway

**Keywords:** COX-2 inhibitor, host-directed therapy, inflammation, innate immunity, macrophages, M*ycobacterium avium*, M*ycobacterium tuberculosis*

## Abstract

**Background:**

Current tuberculosis treatment regimens could be improved by adjunct host-directed therapies (HDT) targeting host responses. We investigated the antimycobacterial capacity of macrophages from patients with tuberculosis in a phase 1/2 randomized clinical trial (TBCOX2) of the cyclooxygenase-2 inhibitor etoricoxib.

**Methods:**

Peripheral blood mononuclear cells from 15 patients with tuberculosis treated with adjunctive COX-2i and 18 controls (standard therapy) were collected on day 56 after treatment initiation. The ex vivo capacity of macrophages to control mycobacterial infection was assessed by challenge with *Mycobacterium avium*, using an in vitro culture model. Macrophage inflammatory responses were analyzed by gene expression signatures, and concentrations of cytokines were analyzed in supernatants by multiplex.

**Results:**

Macrophages from patients receiving adjunctive COX-2i treatment had higher *M. avium* loads than controls after 6 days, suggesting an impaired capacity to control mycobacterial infection compared to macrophages from the control group. Macrophages from the COX-2i group had lower gene expression of *TNF*, *IL-1B*, *CCL4*, *CXCL9,* and *CXCL10* and lowered production of cytokines IFN-β and S100A8/A9 than controls.

**Conclusions:**

Our data suggest potential unfavorable effects with impaired macrophage capacity to control mycobacterial growth in patients with tuberculosis receiving COX-2i treatment. Larger clinical trials are required to analyze the safety of COX-2i as HDT in patients with tuberculosis.

**Clinical Trials Registration:**

NCT02503839.

## Teaser

The study investigated the antimycobacterial capacity of macrophages from patients with tuberculosis in a phase 1/2 randomized clinical trial of the cyclooxygenase-2 inhibitor etoricoxib. Our data suggest potential unfavorable effects with impaired macrophage capacity to control mycobacterial growth after COX-2i treatment.

Tuberculosis caused by *Mycobacterium tuberculosis* has a major impact on global health. In 2021 the World Health Organization reported 10 million new cases of tuberculosis, causing 1.5 million deaths worldwide [1]. Current tuberculosis treatment regimens have several challenges, such as long duration and side effects that complicate patient adherence, which in turn reduce cure rates and fuel antimicrobial resistance [2, 3]. Host-directed therapy (HDT) given adjunctive to standard tuberculosis treatment aims to tailor host immune responses towards more efficient *M. tuberculosis* killing while reducing destructive inflammation, facilitating shorter treatment, higher cure rates, and better-preserved tissue architecture [4, 5]. Anti-inflammatory drugs such as cyclooxygenase inhibitors (COX-is) have been suggested as a potential HDT strategy [6]. Globally, COX-is are already used in clinical practice to treat tuberculosis symptoms and side effects of treatment, but there are limited data on the effects of COX-is on immune responses during *M. tuberculosis* infection in humans and their role as adjunctive HDT [7].

Cyclooxygenase 2 (COX-2) is a rate-limiting enzyme in the production of prostanoids, among which prostaglandin E2 (PGE2) is an important immunomodulator in tuberculosis [8–10]. The role of PGE2 may depend on the stage of *M. tuberculosis* infection, with differential participation during the early and late phases of experimental tuberculosis [9]. PGE2 may modulate both innate and adaptive immune responses, where PGE2 has been shown to confer unfavorable effects by dampening T-cell responses in chronic stages of *M. tuberculosis* infection, partly by inducing regulatory T cells [9–12]. However, findings also suggest that PGE2 has a critical role in the early response by acting in concert with interleukin 1β (IL-1β) in limiting the development of detrimental type 1 interferons (IFNs) and by regulating macrophage function [13].

Macrophages are both the first responders and the predominant cell type implicated in *M. tuberculosis* infection but may act as niches for sustained infection [14]. COX-2 expression increases in *M. tuberculosis*-infected macrophages, and one proposed protective effect of downstream PGE2 is to modulate cell death in favor of apoptosis [8, 15, 16]. However, others have found that the reduced ability of macrophages to control mycobacterial infections may be linked to enhanced synthesis of PGE2, arguing that PGE2 suppresses macrophage function [17–19].

In preclinical studies, the effects of COX-2i treatment in *M. tuberculosis* infection seem dependent on timing, local concentrations of PGE2 at the site of infection, infection kinetics, bacterial load, and route of infection [20–22]. However, evidence from clinical trials is lacking. We have recently shown in a randomized clinical trial (TBCOX2) that the COX-2i etoricoxib adjunctive to standard tuberculosis treatment did not affect cellular or humoral immune responses, although the drug was well tolerated with no safety concerns [23]. Here, in a substudy of TBCOX2, we assess macrophages obtained from patients with tuberculosis treated with or without adjunctive COX-2i and explore their capacity to control mycobacterial growth in an in vitro infection model.

## METHODS

### Study Subjects

The participants were recruited from a randomized open-label phase 1/2 clinical trial conducted at Oslo University Hospital, Oslo, Norway (TBCOX2; NCT02503839) in 2015–2019 [23]. Patients aged 18–70 years diagnosed with drug-sensitive tuberculosis receiving standard tuberculosis treatment were randomized into a COX-2i group treated with adjunctive COX-2i (etoricoxib) 120 mg orally, daily for 140 days or receiving standard tuberculosis treatment only (controls). Blood samples were collected at day 56 when all patients in both groups had experienced sputum culture conversion and all patients showed clinical improvement (COX-2i group, n = 15; and controls, n = 18). Characteristics of patients at baseline have previously been described (Supplementary Table 1) [24].

### Ethical Considerations

The TBCOX2 study was approved by the Regional Committees for Medical and Health Research Ethics (TBCOX2 REK SØ 2015/692, EudraCT No. 2014–004986-26). Biobank samples were collected and stored in the Research Biobank Infectious Diseases (Forskningsbiobank Infeksjonssykdommer, REK 1.2006.181-S-0885, SHDNR. 09/513), Department of Infectious Diseases, Oslo University Hospital, Ullevål. Written informed consent was obtained from all participants before inclusion.

### Cell Culture and Macrophage Differentiation

Blood samples were collected in CPT cell preparation tubes (BD Biosciences), using sodium-heparin as anticoagulant, and immediately centrifuged 15 minutes at 1700*g*. Peripheral blood mononuclear cells (PBMCs) were isolated by density gradient and cryopreserved in 20% dimethyl sulfoxide/80% fetal calf serum at −150°C until analysis. PBMCs were thawed and rested in preheated complete medium: Roswell Park Memorial Institute 1640 with 680 μM L-glutamine and 10 mM HEPES (all Gibco) and 10% pooled human serum (blood bank, St Olav’s Hospital) for 1 hour at 37°C and 5% CO_2_. Cells were then counted using a counter (Beckman Coulter), and viability was assessed using trypan blue solution. Most samples had viability >90%, but samples with low viability (<80%) and/or variable cell numbers in wells were omitted from further analyses. Human monocyte-derived macrophages were generated from thawed PBMCs by plastic adherence and differentiation in complete medium supplemented with 10 ng/mL macrophage colony-stimulating factor (M-CSF; Preprotech) for 6 days. From decades of experience with intermittent testing, the monocyte purity is >80%–90% with <5% T cells using plastic adherence, and the additional wash step postinfection further improves purity because lymphocytes are nonadherent. We cannot, however, exclude the presence of contaminating cells that may influence the results, especially gene expression data due to the sensitivity of quantitative polymerase chain reaction (qPCR). Care was thus taken in data analysis to exclude genes not expressed by monocytes.

### Mycobacterium avium Culture, Macrophage Infection, and CFU Determination

*M. avium* strain 104 was cultured in liquid Middlebrook 7H9 medium (Difco/Becton Dickinson) containing 0.2% glycerol, 0.05% Tween 80%, and 10% albumin dextrose catalase. On the day of infection, log-phase bacteria were washed with phosphate-buffered saline (PBS), subjected to 3 cycles of 30-second sonication, and passed through a gauge 15 needle to ensure a single-cell suspension. Macrophages were seeded at 50 000 cells/well in 96-well plates and infected with *M. avium* at a multiplicity of infection of 10 for 10 minutes, washed, and incubated for 2 hours or 6 days. To assess intracellular burden, infected cells were lysed with PBS/0.02% Triton X-100 (Sigma) and plated on 7H10 Middlebrook plates. *M. avium* colony forming units (CFUs) were counted after 21 days.

### Cytokine Multiplex Assay

Supernatants (100 µL) were harvested from human macrophages before, and 4 hours and 6 days postinfection with *M. avium*. Cytokine secretion profiles for IL-18, monocyte chemoattractant protein-1 (MCP-1), IL-8, vascular endothelial growth factor A (VEGF-A), IL-10, S100A8/A9, granulocyte-macrophage colony-stimulating factor (GM-CSF), inducible protein-10 (IP-10), IL-1Ra, IL-1β, IL-1α, IFN-γ, tumor necrosis factor-α (TNF-α), IFN-α, IFN-β, IL-6, and IL-12/IL-23p40 were analyzed. The concentration of cytokines was determined using a custom panel from Procartaplex (Invitrogen, ThermoFisher Scientific) and analyzed with a Luminex 200 instrument (BioRad). Out-of-range values in the upper range were set to the highest measurable concentration and values below blank were set as zero.

### RNA Extraction and Gene Expression Profiling

Total RNA was harvested from macrophages before and 4 hours after in vitro *M. avium* infection and extracted using RNeasy Microkit according to the manufacturer's protocol (Qiagen), which included DNase I digestion (RNase-free DNase set). The concentration of total RNA was measured by spectrophotometry (NanoDrop). Gene expression profiling of host responses was performed using the nCounter Human Inflammation V2 kit (nanoString), which consists of 249 inflammation-related genes and 6 internal reference genes. For each sample, 100 ng of total RNA was analyzed following manufacturer's instructions. Counts data were processed using nSolver software (version 4.0), normalized to 6 reference genes, and expressed as mRNA counts.

### Statistical Analysis

For the mycobacterial CFUs and cytokine multiplex analyses, nonparametric statistical methods were applied, Mann-Whitney *U* test for unpaired groups and Wilcoxon signed rank test for paired groups. Statistical analysis was performed by SPSS statistics 25 (IBM) and Graphpad Prism 8 (Graphpad Software, Inc). Significance was set to *P* < .05. For gene expression, the raw counts were normalized as previously described [25] using negative controls, positive controls, and housekeeping genes via nSolver version 4.0 software (nanoString), and the Advanced Analysis 2.0 plugin (nanoString) where default settings automatically set normalization probes by using the geNorm algorithm. Analysis was performed with the use of XQuartz 2.8.1 [25]. Normalization for run-to-run and sample-to-sample variability was done by dividing counts within a lane by the geometric mean of the reference/normalizer probes from the same lane. To calculate the differential expression of genes, default settings using the optimal method in the advanced analysis 2.0 plugin (nanoString) were used. Briefly, a negative binomial mixture model was applied for low-expression probes and a simplified negative binomial model for high-expression probes. In situations where the algorithm was not converging, the linear regression method was used instead [26]. Given the number of simultaneous tests and an expected increase in type 1 error, false positives were controlled for using the Benjamini-Yekutieli false discovery rate, resulting in adjusted *P* values as referred in the “Results” section. Figures were generated with GraphPad Prism 8 (GraphPad Software, Inc).

## RESULTS

### Reduced Mycobacterial Clearance in Macrophages From Patients With Tuberculosis Treated With the COX-2i Etoricoxib

**Figure 1.**
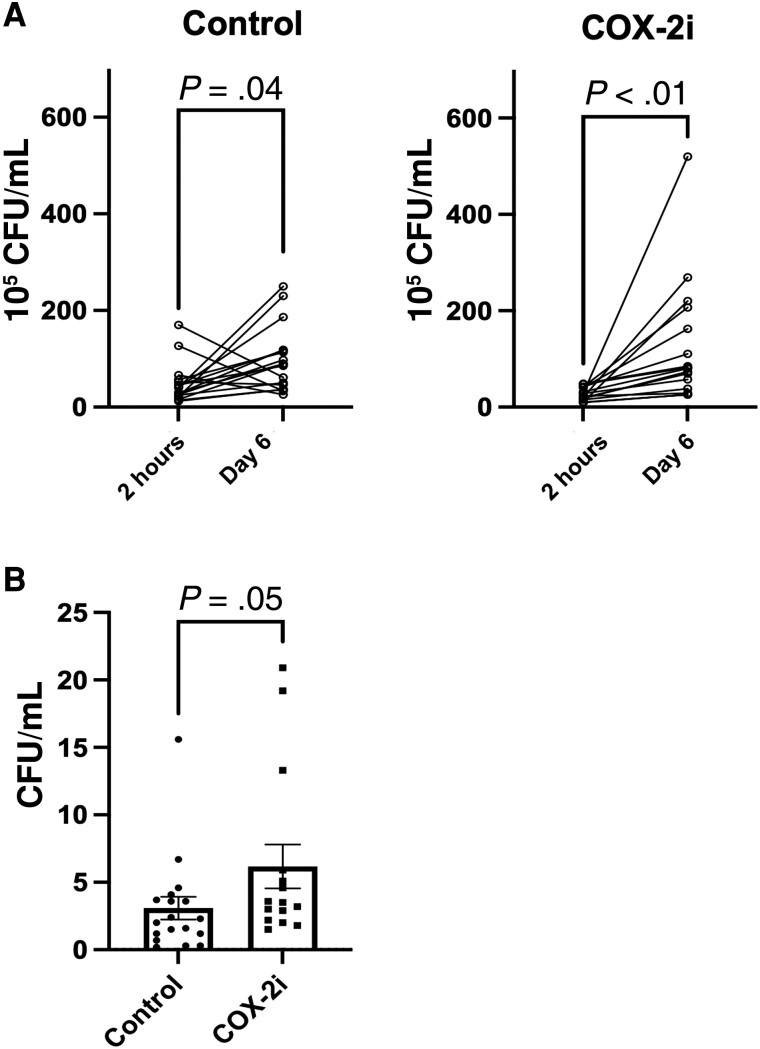
Reduced mycobacterial clearance in macrophages from patients with tuberculosis treated with the adjunctive COX-2i etoricoxib. Macrophages from patients with tuberculosis treated with adjunct COX-2i (n = 15) or standard therapy only (controls, n = 18) were infected with *Mycobacterium avium* and the intracellular bacterial burden was compared after 6 days by assessing the number of colony-forming units (CFUs). *A*, CFU counts for uptake (2 hours postinfection) and 6 days postinfection are compared for each group: control (*P* = .04) and COX-2i (*P* < .01). Differences were calculated using the Wilcoxon signed rank test for paired samples. *B*, Ratio of *M. avium* intracellular burden day 6 relative to 2 hours postinfection. The COX-2i group shows elevated CFU counts (*P* = .05) compared to controls. Data are plotted as mean ± SD. Differences were calculated using the Mann-Whitney *U* test for unpaired samples.

Macrophages are the primary host cell of *M. tuberculosis*, and their degree of bacterial control determines the infection trajectory and overall disease outcome [14]. To evaluate if in vivo etoricoxib treatment affected the microbicidal capacity of macrophages, we infected macrophages in vitro with *M. avium* and compared the intracellular bacterial burden after 6 days by assessing the number of CFU. To account for possible variation in the cell number seeded, we normalized day 6 CFUs (growth) to their respective 2-hour postinfection CFUs (uptake). *M. avium* replicated significantly within macrophages from both patient groups over the 6 days of infection (controls, *P* = .04; COX-2i group, *P* = .001; Figure 1*A*). Interestingly, macrophages from patients in the COX-2i group had higher CFUs (*M. avium* loads) than controls, indicating a reduced ability to control mycobacterial infection (*P* = .05; Figure 1*B*).

### Adjunctive COX-2i Treatment of Patients With Tuberculosis Lowers the Basal Expression of Inflammatory Genes in Macrophages

**Figure 2.**
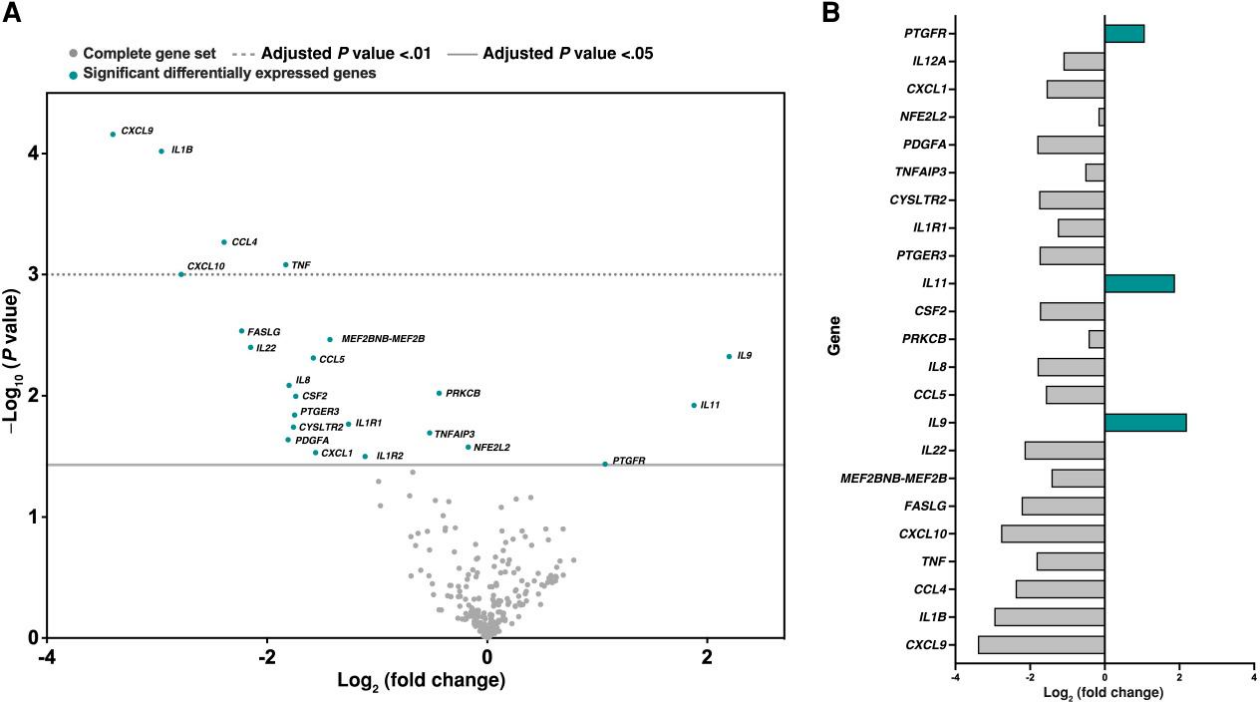
Reduced basal expression of inflammatory genes in macrophages following adjunctive COX-2i treatment. Inflammatory gene expression of 249 targets in macrophages from patients with tuberculosis on standard tuberculosis treatment with or without adjunctive COX-2i (COX-2i group, n = 15; controls, n = 18) obtained by nanoString analysis. *A*, Comparison of gene expression in uninfected macrophages between the COX-2i group and controls (the latter shown as baseline). Volcano plot displaying each gene's −log_10_ (*P* value) and log_2_ fold change. Horizontal lines indicate various false discovery rate thresholds or *P* value thresholds if there is no adjustment to the *P* values. Genes with adjusted *P* value ≤ .05 are shown with label and color. Differentially expressed genes were identified using simplified negative binomial models, mixture negative binomial models, or log-linear models based on the convergence of each gene using the advanced analysis 2.0 plugin (nanoString). *B*, Graph illustrating log_2_ fold changes for the most significantly differentially expressed genes. Grey and green bars represent down- and upregulation, respectively.

Macrophages with a proinflammatory phenotype are shown to have better control of mycobacterial infections [27–29]. To investigate why macrophages from patients receiving COX-2i had lowered microbicidal capacity, we explored the basal inflammatory gene expression profiles of uninfected macrophages by NanoString analysis including 249 inflammation-related genes. A global analysis of changes in genetic pathways in the COX-2i group versus the control group (standard tuberculosis treatment only) at baseline and after *M. avium* infection (4 hours) are shown in Supplementary Figure 1*A*. The pathway scoring of genes showed that inflammatory pathways such as the C-type lectin receptor pathway, the IL-1 signaling pathway, and the nuclear factor-κB (NF-κB) pathway were lower in the COX-2i group at baseline, but the COX-2i group response to *M. avium* infection was stronger in several inflammatory pathways, although none of these global changes were statistically significant. Analyses of individual genes revealed that uninfected macrophages in the COX-2i group presented a significantly lowered expression of *CXCL-9* (*P* = .01), *IL-1β* (*P* = .01), *CCL4* (*P* = .04), *TNF* (*P* = .04), and *CXCL-10* (*P* = .049) compared to controls (Figure 2*A* and 2*B*). In COX-2i–treated subjects, we also observed a trend of reduced levels of 33 proinflammatory genes, albeit not significant (Figure 2*A*). Overall, this suggests that in vivo COX-2i treatment reduces macrophage inflammatory capacity with possible negative effects for mycobacterial containment and killing.

**Figure 3.**
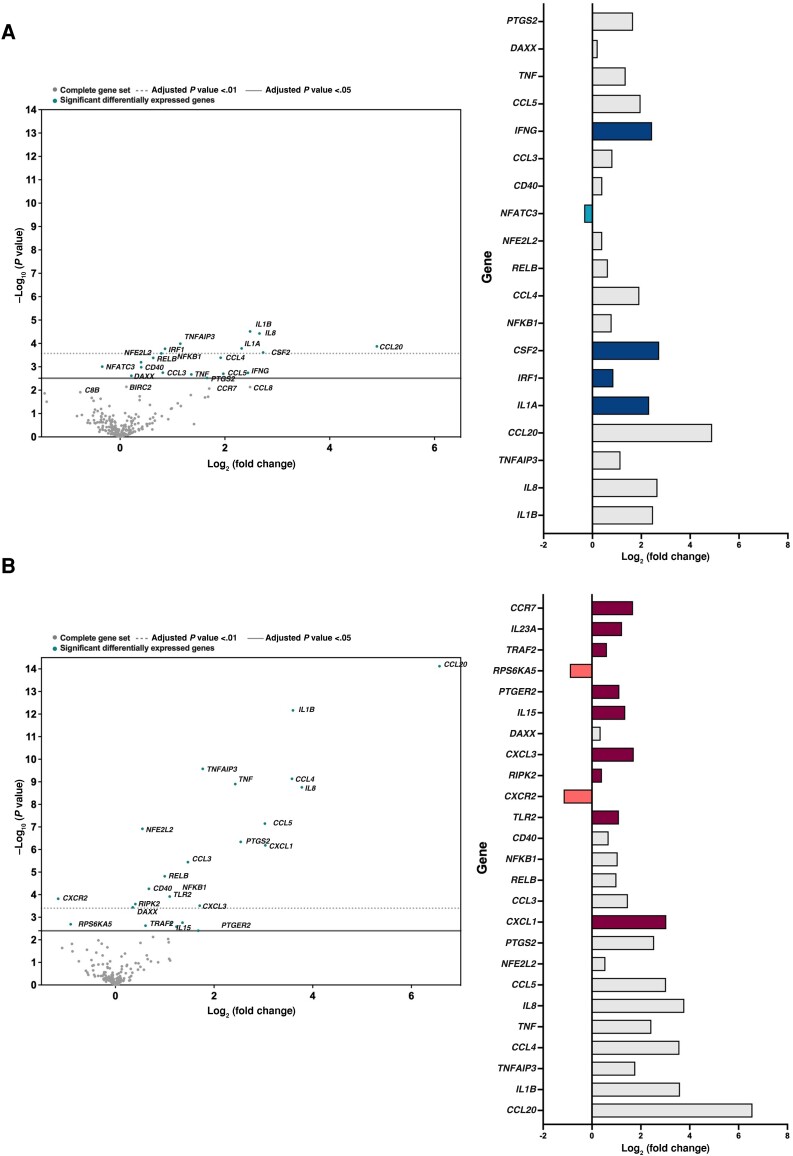
Changes in inflammatory gene expression in macrophages following *Mycobacterium avium* infection. Inflammatory gene expression of 249 targets in macrophages from patients with tuberculosis treated with adjunct COX-2i (n = 15) or standard chemotherapy only (controls, n = 18) 4 hours postinfection with *M. avium* (nanoString analysis). Comparison between uninfected and infected macrophages within the controls (*A*) and the COX-2i group (*B*). Volcano plots (left) display each gene's −log_10_ (*P* value) and log_2_ fold change. Horizontal lines indicate *P* value thresholds. Genes with adjusted *P* value ≤ .05 are shown with label and color. Histogram plots (right) illustrate log_2_ fold changes for the most significantly differentially expressed genes. Grey bars indicate genes that were upregulated in both groups; dark blue bars and maroon bars indicate genes that were upregulated only in the control and Cox-2i group, respectively; light blue and orange bars indicate genes that were downregulated only in the controls and Cox-2i group.

We next compared changes in gene expression in macrophages 4 hours after *M. avium* infection. A significant upregulation of the following genes was seen after *M. avium* infection in both patient groups: *IL-1β, IL-8, TNFAIP3, CCL20, NFKB1, CCL4, RELB, NFE2L2, CD40, CCL3, CCL5, TNF, DAXX,* and *PTGES2* (Figure 3*A* and 3*B*). By contrast, *IL-1α, IRF-1, CSF-2,* and *IFN-γ*, were significantly upregulated in controls, but not in the COX-2i group (Figure 3*A*). Conversely, the following genes were significantly upregulated in the COX-2i group, but not in controls: *CXCL1, TLR2, RIPK2, CXCL3, IL-15, PTGER2, TRAF2, IL-23,* and *CCR7* (Figure 3*B*). However, despite the lower basal expression of inflammatory genes in macrophages from COX-2i–treated patients, and the differential upregulation of genes within the 2 study arms, there were no significant differences in gene expression between the groups after infection (Supplementary Figure 1*B*).

### Adjunctive COX-2i Treatment Lowers the Secretion of Inflammatory Cytokines by Macrophages Infected Ex Vivo With M. avium

**Figure 4.**
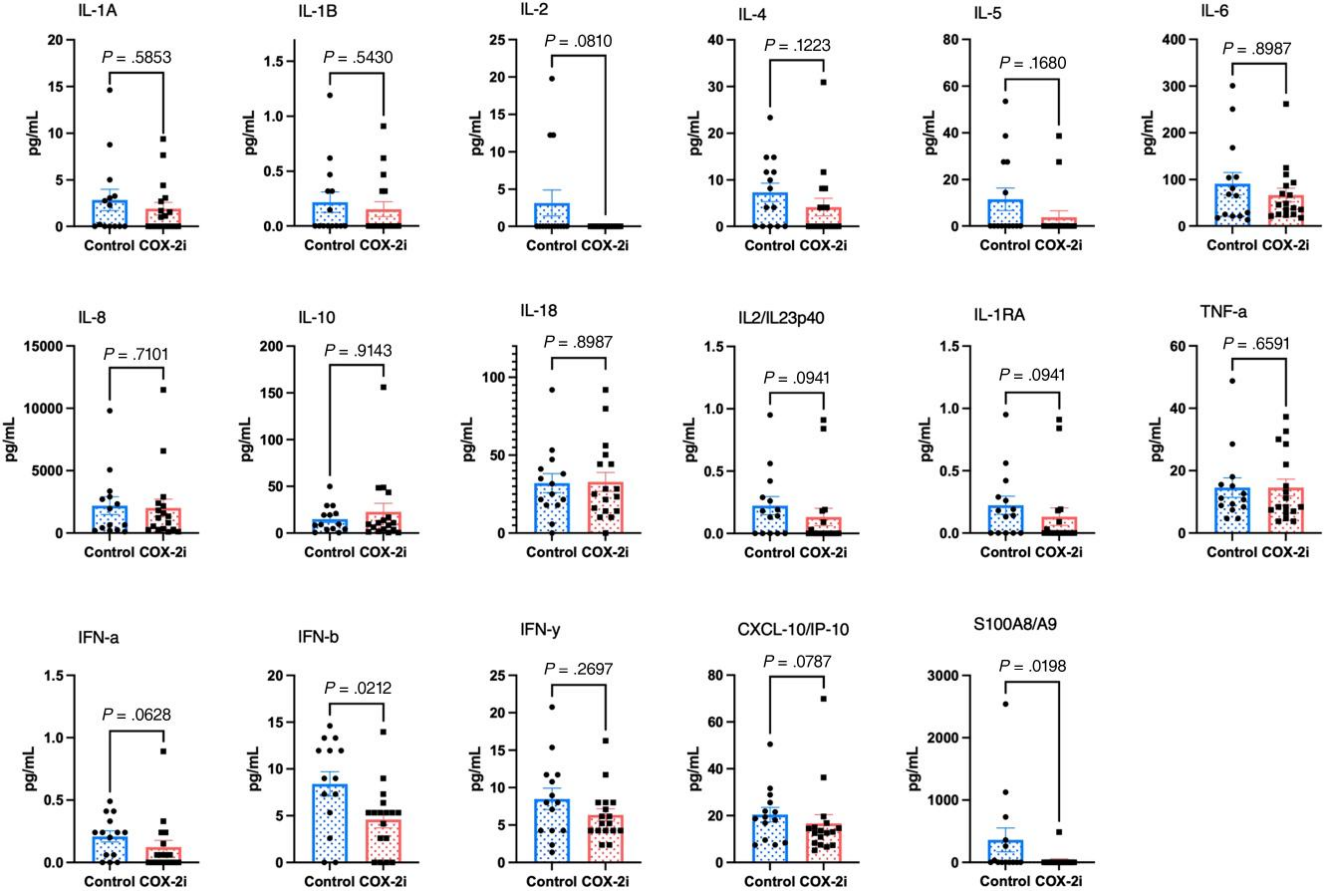
Adjunctive COX-2i treatment of patients with tuberculosis lowers the secretion of inflammatory cytokines by macrophages infected with *Mycobacterium avium* ex vivo. Culture supernatants were harvested from patient macrophages (COX-2i–treated group, n = 15; controls, n = 18) 4 hours after *M. avium* infection, and cytokine concentrations were analyzed. Differences were calculated using the Mann-Whitney *U* test for unpaired samples. Abbreviations: CXCL-10, C-X-C motif chemokine ligand 10; IFN, interferon; IL, interleukin; IP-10, inducible protein-10.

To investigate if the patterns of gene expression were reflected in macrophage cytokine production, the concentration of central cytokines and inflammatory mediators in cell culture supernatants 4 hours and 6 days after *M. avium* infection were measured. After 4 hours, levels of IFN-β and the alarmin S100A8/A9 (calprotectin) were significantly reduced in the COX-2i group compared to controls (Figure 4). Likewise, we observed a trend of reduced concentration of IL-2, IL-4, IFN-α, CXCL-10, IL-12/IL-23p40, and IL-1Ra in the COX-2i group compared to controls (Figure 4). However, at 6 days postinfection no significant differences in macrophage cytokine production between the patient groups were observed (Supplementary Figure 2).

## DISCUSSION

COX-2is are suggested candidates for HDT to alleviate harmful and excessive inflammatory responses in *M. tuberculosis* infection [6] and to improve mycobacterial control. In the present study, we explored the effects on macrophages of the COX-2i etoricoxib given in adjunctive to standard tuberculosis treatment in the context of the clinical trial TBCOX2 [23]. We show that treatment with etoricoxib for 2 months reduces the macrophage capacity to control a second mycobacterial infection in vitro. In addition, uninfected macrophages from COX-2i–treated patients showed an overall lower inflammatory response with a reduced basal expression of *CXCL9, IL-1β, CCL4, TNF,* and *CXCL10* and, shortly after *M. avium* infection, reduced production of IFN-β and S100A8/A9, compared to controls. While all patients in our study experienced clinical improvement and sputum conversion within 56 days of standard tuberculosis treatment, our findings suggest that macrophages derived from COX-2i–treated patients have reduced mycobacterial control upon reinfection.

Our findings indicate that COX-2 inhibition imprints changes of reduced inflammatory and antimycobacterial capacity in macrophages that are sustained even upon ex vivo cultivation. This links to the newly emerged concept of innate memory, involving epigenetic and metabolic reprogramming of innate immune cells, which changes the responsiveness to subsequent challenges [30]. Importantly, in our study, we reinfected macrophages derived from blood monocytes from patients with tuberculosis treated with or without adjunctive COX-2i. Consequently, our findings of reduced mycobacterial control and reduced basal expression of inflammatory genes in macrophages from the COX-2i–treated patients may imply that the effect of adjunctive COX-2i treatment is imprinted in circulating blood monocytes. This imprint lasts through the ex vivo differentiation into macrophages and changes their inflammatory profile and antibacterial capacity to a second, ex vivo mycobacterial challenge. Although, there is evidence of trained innate immunity in tuberculosis, with increased responsiveness to subsequent challenges [31], to our understanding, there are no other studies showing the potential of COX-i in mediating lasting memory in innate immune cells in *M. tuberculosis* infection. We acknowledge that our observation of a possible innate memory mechanism is a hypothesis-generating observation, and warrants further investigation.

Our study is the first to investigate mycobacterial killing capacity of ex vivo macrophages after in vivo COX-2i treatment in patients with tuberculosis [32]. Preclinical studies present conflicting results regarding the use of COX-2i in tuberculosis [6, 20, 21]. In animal studies, high levels of PGE2 in chronic phases of infection (eg, >45 days postinfection) have been found to inhibit phagocytosis and bacterial killing, reduce the production of reactive nitrogen species, and limit Th1 cytokines [9, 11]. Thus, inhibition of PGE2 at this stage might favor host control [9, 17]. Vilaplana et al showed in a murine intravenous *M. tuberculosis* infection model that COX-i could alleviate excessive inflammation, resulting in improved outcome [21]. While confirming this finding in a similar mouse model, Mortensen et al further showed that treatment with COX-2i increased mycobacterial burden in a low-dose aerosol mouse model, possibly by reducing the protective capacity of CD4 T cells [20]. This is in line with our recently published phase 1/2 randomized trial, where we show that although the patients with tuberculosis experienced clinical improvement and sputum conversion, adjunctive COX-2i with etoricoxib did not improve *M. tuberculosis*-specific cellular or humoral responses compared to controls [23]. Inconsistent reports of COX-2is are further reflected in in vitro studies, where some report that COX-2 accelerates mycobacterial elimination [11, 33], while others find that PGE2 levels are inversely correlated with the macrophage capacity to control mycobacterial infections [11, 34, 35].

In the current study, we found that uninfected macrophages from COX-2i–treated patients had significantly lower gene expression of *TNF, IL-1β, CCL4, CXCL9*, and *CXCL10,* compared to controls. These genes are important in antimycobacterial defense and reduced expression may be linked to the impaired control of mycobacterial growth by macrophages derived from COX-2i–treated patients. TNF-α is critical for tuberculosis control and involved in numerous processes such as macrophage activation, granuloma formation, and restriction of intracellular mycobacterial growth [36, 37]. However, excessive production may lead to extensive tissue damage and clinical deterioration in advanced disease [37]. IL-1β is essential in the host defense against *M. tuberculosis,* illustrated by increased susceptibility to *M. tuberculosis* infection in IL-1β knock-out mice. The chemokines CXCL-9 and CXCL-10 are considered necessary for optimal granuloma formation and bacterial containment [38–40].

To evaluate the impact of COX-2i on the macrophage inflammatory capacity, we analyzed gene expression and secretion of central cytokines in macrophages after 4 hours of *M. avium* infection. Several genes were significantly upregulated in both groups. Interestingly though, *IL-1α, IRF-1, CSF2,* and *IFN-γ* were significantly upregulated only in controls. Furthermore, macrophages from controls had significantly higher levels of IFN-β and the alarmin S100A8/A9 compared to COX-2i–treated patients. The relevance of the observed differences in gene expression and cytokine secretion between the groups is strengthened by our observation of reduced mycobacterial clearance in macrophages from COX-2i–treated patients. The absence of IL-1α has been shown to increase the bacterial burden and worsen lung inflammation in mouse models [13, 14] and IFN-γ is a well-known macrophage activation factor stimulating antimycobacterial activities [14]. On the other hand, increased levels of S100A8/A9 and IFN-β may be unfavorable for the host response due to the reported associations with reduced *M. tuberculosis* clearance [13, 41–43]. Serum S100A8/A9 is elevated in tuberculosis progressors and severe/chronic disease and mediates increasing neutrophil infiltration [41], whereas high and/orsustained levels of type I IFNs are reported to have multiple unfavorable effects during tuberculosis including induction of anti-inflammatory IL-10 and IL-1Ra, inhibition of protective Th1 responses, and promotion of macrophage cell death [12, 42, 43].

Differences in cytokine production between controls and COX-2i–treated patients were not reflected in gene expression data following 4 hours of *M. avium* infection, which might be caused by different kinetics of mRNA and protein levels [44]. However, we observed no differences in cytokine production 6 days after *M. avium* infection. At 6 days postinfection feedback loops and different bacterial loads will influence cytokine levels, and one possible explanation could be that the increased mycobacterial load outweighs the effect of COX-2i. Hence, the effects of COX-2i are difficult to interpret due to the complex interaction between the expressed genes and cytokines measured. However, our findings of reduced capacity of macrophages to control *M. avium* infection, in addition to reduced expression of proinflammatory genes and cytokines, may indicate autoparacrine pathways stimulating antimicrobial defense mechanisms in macrophages, as shown previously in studies from healthy blood donors [45]. However, although both groups showed upregulated gene expression following 4 hours of in vitro *M. avium* infection, macrophages from controls and the COX-2i–treated patients had comparable mRNA levels of inflammatory genes. This implies that other mechanistic explanations than kinetics may also be relevant to explain the reduced control of mycobacterial growth by macrophages from COX-2i–treated patients.

There are limitations to our study and several of the findings should be pursued in future studies to clarify underlying mechanisms. First, a low number of participants in the TBCOX2 trial necessitates careful interpretation of data. Second, we investigated the effects of COX-2i on cells obtained from peripheral blood and not from the actual sites of infection. Also, due to limited number of patient samples and cells per sample, analyses of adaptive immune cells or exploration of possible mechanisms underlying the COX-2i effect on macrophages, such as in vitro supplementation of PGE2 or epigenetic analyses, were not performed. Furthermore, due to logistical concerns, we infected macrophages with *M. avium* and not *M. tuberculosis*. While bacterial uptake was comparable between the patient groups, we did not investigate macrophage processes important to mycobacterial control, such as phagosome maturation, regulation of cell death pathways (apoptosis or necrosis), metabolism, production of reactive oxygen species, or epigenetic changes [14, 46]. Necrotic cell death is proposed to propagate the spread of *M. tuberculosis* [16], but unlike *M. tuberculosis*, *M. avium* lacks the type VII secretion system ESX-1 and does not cause necrotic cell death [29] Thus, by using *M. avium* in our study we may have failed to detect a possible effect of COX-2i on regulating apoptosis versus necrosis. However, we believe that the lowered ability of macrophages to control mycobacterial infection with *M. avium* in our study may be valid also for *M. tuberculosis* [46–48]. Finally, it is possible that our findings are influenced by how the macrophages were differentiated. We differentiated macrophages with M-CSF, which is generally believed to favor M2 polarization, whereas GM-CSF in combination with IFN-γ facilitates M1 macrophages with increased inflammatory and antimycobacterial capacity [49]. However, a study by Lerner et al did not observe major differences in mycobacterial control between human macrophages differentiated with M-CSF versus GM-CSF [50].

In this study, we show potential unfavorable effects of COX-2i on mycobacterial growth. We show that macrophages isolated from COX-2i–treated patients have an impaired capacity to control mycobacterial growth, which may be partly explained by a reduced basal inflammatory capacity. Furthermore, our findings suggest that adjunctive COX-2i imprints a reduced inflammatory capacity in circulating innate immune cells. In conclusion, our study may question the use of long-term adjunctive therapy of COX-2i as part of an HDT strategy to improve tuberculosis immunity and control infection, and further in-depth investigations of microbicidal mechanisms should be conducted.

## Supplementary Data

Supplementary materials are available at *The Journal of Infectious Diseases* online. Consisting of data provided by the authors to benefit the reader, the posted materials are not copyedited and are the sole responsibility of the authors, so questions or comments should be addressed to the corresponding author.

## Supplementary Material

jiad390_Supplementary_Data

## Notes

***Acknowledgments.*** We thank all patients participating in the TBCOX2 study, and the collaborators in the TBCOX2 study at Statens Serum Institut, Denmark (Peter Andersen, Morten Rühwald, Ingrid Kroman, Rasmus Mortensen, and Peter Bang) and Oslo University Hospital, Norway (Dag Kvale, Kjetil Tasken, Kjerstin Røstad, Kjersti Sellæg, Sarah Nur, and Mette Sannes), and researcher Markus Haug at Centre of Molecular Inflammation Research, Trondheim, Norway.

***Financial support*.** This work was supported by the Research Council Of Norway Global Health and Vaccination Research Programme (grant numbers 234493, 223255, and 287696 to T. H. F.); South Eastern Norway Regional Health Authority, Oslo University Hospital, University of Oslo (grant number 2017009); and the Olav Thon Foundation (to T. H. F.).
