# Supplementary material for: The Cyclooxygenase 2 Inhibitor Etoricoxib as Adjunctive Therapy in Tuberculosis Impairs Macrophage Control of Mycobacterial Growth
Source: J Infect Dis. 2023 Sep 18;229(3):888–97. doi: 10.1093/infdis/jiad390 (PMC10938220; doi:10.1093/infdis/jiad390)
Supplement: jiad390_Supplementary_Data [file jiad390_supplementary_data.zip › Supplementary_Table_1.docx]

|  | Total (n = 39*) | COX-2i (n = 18*) | Control (n = 21*) |
| --- | --- | --- | --- |
| Age (median) | 27 (18-52) | 29 (19-49) | 26 (18-52) |
| Male (%) | 21 (54) | 9 (50) | 12 (57) |
| **Clinical presentation** |  |  |  |
| Pulmonary | 28^a^ | 14^b^ | 16^c^ |
| Cavity | 9 | 5 | 4 |
| Extrapulmonary | 7 | 4 | 3 |
| **Symptoms** |  |  |  |
| Cough (%) | 20 (51) | 10 (56) | 10 (48) |
| Night-Sweat (%) | 18 (46) | 7 (39) | 11 (52) |
| Weight loss (%) | 15 (39) | 9 (50) | 6 (29) |
| Fever (%) | 9 (23) | 5 (28) | 4 (19) |
| Chest pain (%) | 11 (28) | 4 (22) | 7 (33) |
| Low:high symptom score^d^ | 17:22 | 8:10 | 9:12 |
| **Findings** |  |  |  |
| BMI^e^ (min-max) | 21 (16-30) | 21 (16-30) | 21 (17-27) |
| ML ratio^f^ (min-max) | 0.33 (0.13-1.36) | 0.36 (0.13-1.36) | 0.33 (0.17-1.4) |
| ESR^g^ (mm/hour, min-max) | 20 (1-116) | 26 (2-105) | 20 (1-116) |
| TTP^h^ (min-max) | 12.2 (2.7-42.1) | 12.8 (2.71-24.9) | 12.2 (4.7-42.1) |
| Ct value^i^ (min-max) | 36 (31-46) | 34 (31-46) | 41 (31-46) |
| *6 samples (3 from the COX-2i group and 3 from the control group) were excluded due to low viability (<80 %) and/or variable cell numbers. The total count for final analysis was n = 33(COX2i, n =15 and Control, n=18).  ^a^4/28, ^b^2/14 and ^c^2/16 with both PTB and EPTB.  ^d^High =>2 of the following symptoms: Cough, night sweat, weight loss and fever. Low = 1 symptom or asymptomatic.  ^e^Body Mass Index  ^f^Myeloid:lymphocyte ratio  ^g^Erythrocyte sedimentation rate  ^h^Time to *Mtb* positive culture, days  ^i^Cycle threshold value | | | |
