# Supplementary figures and images for: The Cyclooxygenase 2 Inhibitor Etoricoxib as Adjunctive Therapy in Tuberculosis Impairs Macrophage Control of Mycobacterial Growth
Source: J Infect Dis. 2023 Sep 18;229(3):888–97. doi: 10.1093/infdis/jiad390 (PMC10938220; doi:10.1093/infdis/jiad390)

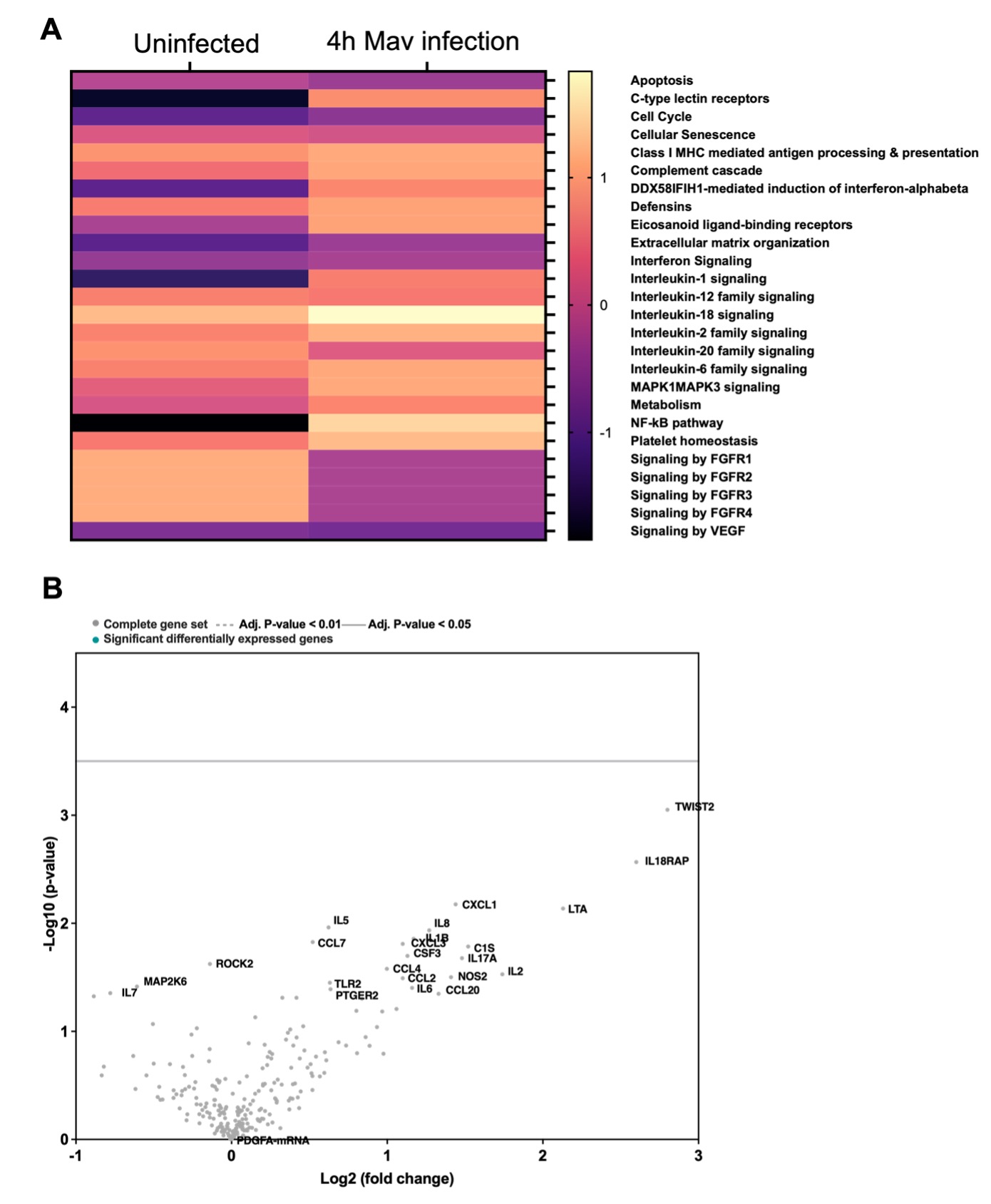

Supplement: jiad390_Supplementary_Data [file jiad390_supplementary_data.zip › Supplementary_Figure_1.jpg]

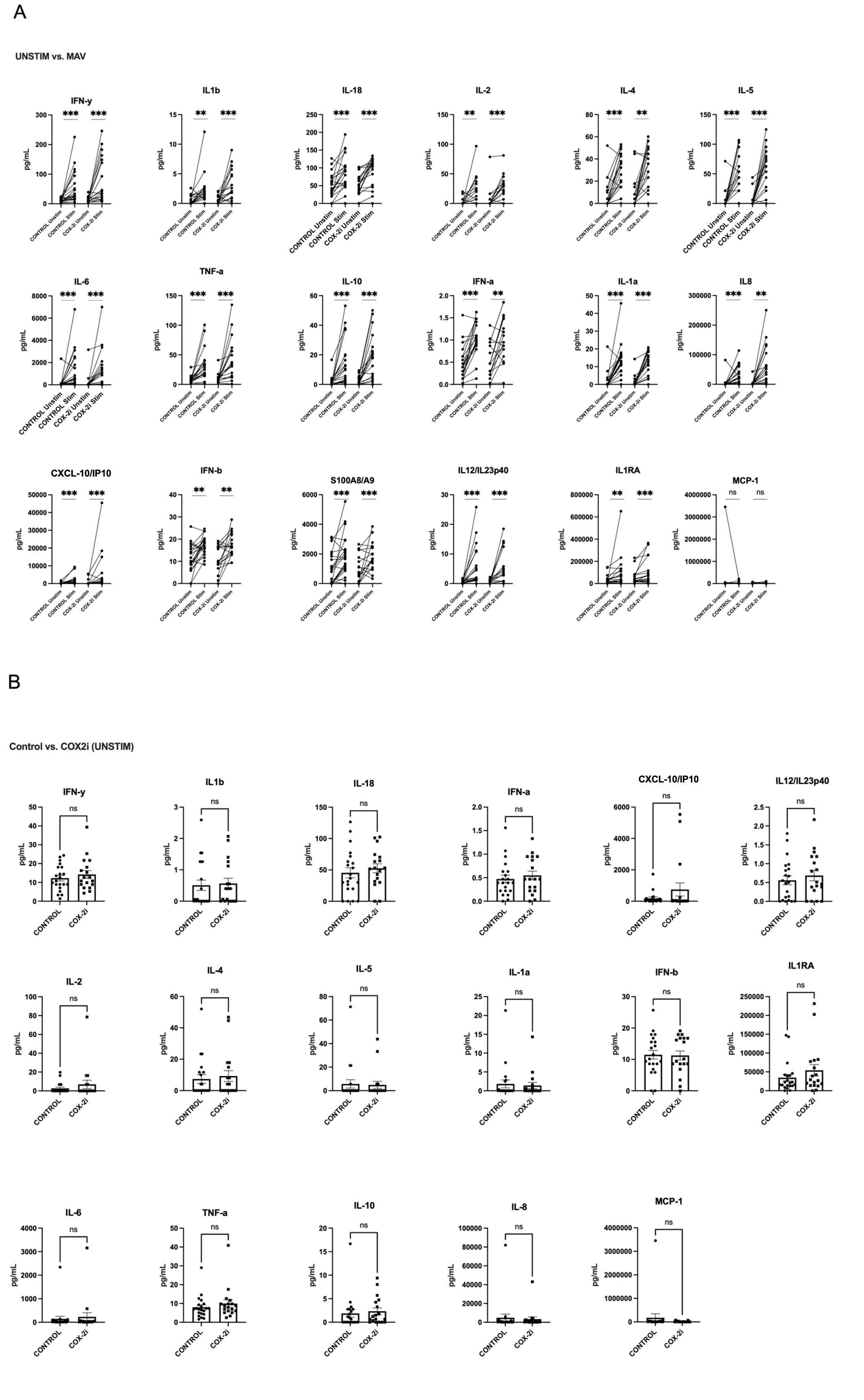

Supplement: jiad390_Supplementary_Data [file jiad390_supplementary_data.zip › Supplementary_Figure_2.jpg]
